# Risk aversion and HIV/AIDS: Evidence from Senegalese female sex workers

**DOI:** 10.1016/j.socscimed.2020.113020

**Published:** 2020-07

**Authors:** Aurélia Lépine, Carole Treibich

**Affiliations:** a University College London, Institute for Global Health, London, UK; b University Grenoble Alpes, CNRS, INRAE, Grenoble INP, GAEL, 38000, Grenoble, France

## Abstract

HIV/AIDS is the second cause of mortality globally and there are 5000 new infections each day. Globally, sex workers are 13 times more at risk of HIV than the general population and in Senegal they have an HIV prevalence 16.5 times greater. Therefore, it is urgent to encourage behaviour change, which requires a better understanding of the reasons why sex workers engage in risky behaviours. We provide new evidence of the role of risk preferences on sexual behaviours, health behaviours and health outcomes of 600 female sex workers in Senegal in July and August 2017. We measure risk aversion of sex workers using an incentivised Gneezy and Potters task in addition to specific risk-taking scales in four domains (in general, finance, health and sex). Understanding of the experimental task was high despite low literacy level of participants. Using ordinary least squares, we find that risk aversion is an important predictor of sex workers’ sexual behaviours. We find that sex workers with higher level of risk aversion have less sex acts with clients, have less clients at risk of HIV, are more likely to engage in protected sex acts and as a result earn less money per sex act. Furthermore, we find that sex workers exhibiting higher level of risk aversion are less likely to be infected with sexually transmitted infections. Results highlight that some associations between risk preferences and sexual and health behaviours are domain specific. To conclude, our results confirm the role of risk preferences in the spread of HIV/AIDS epidemic and suggest the importance of collecting information on self-reported risk aversion to identify individuals who are at a greater risk of HIV/AIDS. Finally, our results provide some rationale in using lottery-based financial incentives to prevent sexually transmitted infections and HIV/AIDS among high-risk populations.

## Highlights

• We study the role of risk preferences on HIV/AIDS among sex workers in Senegal.
• Risk preferences were elicited in a lab-in- the-field experiment and self-reported.
• Risk aversion is a main predictor of sexual behaviours.
• The relationship between risk aversion and behaviours is domain-specific.

## 1 Introduction

The HIV/AIDS burden undermines efforts to reduce poverty and inequalities and to preserve human capital. While the HIV/AIDS epidemic has harshly hit numerous Eastern and Southern African countries, such as Bostwana, Lesotho or Swaziland where the prevalence of HIV/AIDS exceeded 20% in 2012, West Africa has experienced much lower rates of prevalence, current figures ranging from around 4% in Cameroon and Gabon to less than 1% in Senegal, Mauritania and Niger (UNAIDS, 2017). In Western African countries, the epidemic is concentrated among high-risk groups. For instance, female sex workers in Senegal are up to 16.5 times more likely to be infected with HIV/AIDS than the general population with an HIV/AIDS prevalence of 6.6% and a high prevalence of other sexually transmitted infections (STIs) (54.9% for vulvovaginitis, 10.7% for syphilis, 5.8% for trichomoniasis) (APAPS and IRESSEF, 2015). These figures are particularly alarming since the presence of any STI increases both the risk of new infections among HIV-negative people and the risk of transmission from HIV-positive people (Galvin and Cohen, 2004; Wasserheit, 1992) In addition, the nature of the work of commercial sex workers leads to high rates of transmission to the general population through clients’ infection. There is evidence globally that targeting high-risk groups, such as sex workers, in low prevalent concentrated epidemics translates into HIV reduction among the general population (Vassall et al., 2014). Epidemiological models suggest that in West Africa, 75% of HIV infections among men are attributable to sexual intercourse with sex workers and that an elimination of HIV risk associated with sex work would eradicate the heterosexual HIV epidemic (Alary and Lowndes, 2004; Alary et al., 2013)

Our paper focuses on Senegal, the only African country that legalised and regulated sex work with a public health intervention. Since 1969, Senegalese female sex workers aged more than 21 years old have been compelled to register with a health centre and to attend routine health visits in order to be tested and treated for STIs and to receive free condoms (Chersich et al., 2013) Although sex work regulation is effective in reducing STIs through compulsory health visits, registration does lead to an increase in risky behaviours. In a recent evaluation of the registration policy, it was found that registration made sex workers more willing to solicit clients in bars and nightclubs, which was associated with riskier clients and riskier sexual behaviours (Ito et al., 2018). Therefore, it is urgent to encourage behaviour change, which requires a better understanding of the reasons why sex workers engage in risky health and sexual behaviours.

Risk preferences is defined as the extent to which people are willing to take risk. There is robust evidence in the literature that risk preferences strongly influences economic decisions (Benartzi and Thaler, 1999; Cohn et al., 1975; Guiso et al., 1996). Other studies also documented that risk preferences are associated with health behaviours (Anderson and Mellor, 2008; Szrek et al., 2012) and prevention activities (Goldzahl, 2017; Picone et al., 2004). Our study focuses on sexual and health behaviours of sex workers and an interesting feature of those behaviours is that risky health behaviours of sex workers receive a positive income premium. In fact, there is strong evidence that sex workers engage in riskier sex acts in order to increase sex work revenues. Several studies have estimated a large positive premium for riskier sex acts such as unprotected sex acts (Arunachalam and Shah, 2013; Gertler et al., 2005; Rao et al., 2003). As such, sex worker's likelihood of engaging in risky sexual behaviours depends on their preference for health relatively to wealth (Ito et al., 2018). However, so far there is no evidence of the role of risk preferences on behaviours of sex workers. The identification of the role of risk preferences in the HIV/AIDS epidemic could be useful to policy-makers and NGOs working with high-risk groups that could consider this parameter when designing public health interventions. For instance, the identification of high-risk populations exhibiting higher risk preferences level could improve the allocation of HIV resources. In addition, a better understanding of the role of risk preferences on HIV transmission could allow to improve the design of HIV prevention policies. Recent evidence suggests that, for instance, the use of lottery-based financial incentives can be an effective intervention to reduce STIs in high-risk populations (Björkman Nyqvist et al., 2018).

In order to test whether risk preference is a personality trait that has an important role to play in the spread of HIV/AIDS, we collected socio-economic, psychological and biological data from female sex workers living in Dakar. Precisely, we conducted a survey in 2017 among 592 sex workers to analyse the effect of risk preferences on behaviours. Our main result indicates that risk preferences are a main driver of sexual behaviours of sex workers. We found that risk averse sex workers demand more HIV prevention services, have fewer clients and are much more likely to engage in safer sex acts.

Our paper contributes to the literature of the role of risk preferences on sexual behaviours, health behaviours and health outcomes. So far, ambiguous results regarding the role of risk preferences on health behaviours have been obtained in the literature. For instance, (2008) obtained a negative correlation between risk aversion and behaviours such as smoking, heavy drinking, seat-belt use and obesity using the Holt and Laury (2002) lottery choice experiment, but Szrek et al. (2012) did not find any association between these behaviours and risk aversion. Szrek et al. (2012) considered three other risk preferences measures (the Dohmen et al. (2011)'s self-reported scale, DOSPERT scale (Weber et al., 2002) and the balloon analogue risk task (BART)) and pointed that the measures that predicted the best actual behaviour was the general one-item Dohmen measure, while no correlation was detected between health behaviours and the BART measure. However, the BART measure was also used by Lejuez et al. (2002) in a laboratory experiment on 86 participants, who on the contrary, found that risk aversion was negatively correlated with risky sexual behaviours. A few studies investigated the relationship between prevention activities such as screening (Goldzahl, 2017; Picone et al., 2004) and found that less risk averse individuals were more likely to undergo testing.

Finally, Lammers and Van Wijnbergen (2007) also found a positive relationship between risk preferences, HIV status and the perception of being infected with HIV among 163 South African students.

Unlike these previous studies, we investigate the relationship between risk preferences and health by focusing on sex workers, a group at high-risk of HIV and whose risky behaviours with their clients and their sexual networks are the major contributor to overall HIV transmission rates in Senegal. To our knowledge, this is the first study that investigates the role of risk preferences among sex workers and in addition, this is one of the largest lab-in-the-field experiments measuring risk preferences using both experimental and self-reported measures in a low-income country setting. The fact that our measures of risk aversion are domain-specific allows us to explore potential domain specific relations between risk preferences and sexual or health behaviours and health outcomes.

The remainder of the paper is organized as follows. In Section 2, we present a theoretical framework justifying the introduction of risk aversion in different domains. Section 3 presents the experimental set up and methods to measure risk preferences. Section 4 displays the empirical specification. Data and risk aversion measures are presented in Section 5, 6 respectively. Finally, results and a series of robustness checks are presented in Section 7 and discussed in Section 8.

## 2 Theoretical framework

Let's consider a sex worker who derives utility from her health status and her consumption level, reflected by her income.

In period 1, the sex worker has a level of health *H*. Let's assume that she can choose to engage or not in risky sexual behaviour in period 1. On the one hand, if she engages in safe sex, she earns an income *I* and does not contract any disease in period 2, which enables her to maintain her level of health and consumption. On the other hand, if she engages in risky sex, she earns more income in period 1 ($I^{+}>I$) but faces a probability *p* of being infected with a STI in period 2. In that case, she will have a lower level of health $H^{-}<H$ and will lose part of her income $I^{-}<I$.

Let's assume a discount rate β between the two periods and that the utility function U(I,H) is an additively separable function. As pointed out in the literature (Evans and Viscusi, 1991; Finkelstein et al., 2009, for instance), preferences may not be separable in practice, i.e. the individual's marginal utility of income may differ when her health improves or deteriorates. We make the separability assumption here for tractability so as to illustrate, in a clear cut case, that individuals may have different attitudes towards risk regarding health and towards risk regarding income, and why this might affect their behaviour. The same type of conclusions could be reached for non-separable preferences, but would be more difficult to present in a simple fashion: U(I,H)=v(I)+w(H).

In order to decide whether to engage or not in risky sex, the sex worker compares her utility in both cases. Equations (1), (2)) present the individual expected utility if she engages in safe sex, $E_{S}\left(u\right)$, and if she opts for the risky behaviour, $E_{R}\left(u\right)$, respectively.

(1) $$E_{S}\left(u\right)=U\left(I,H\right)+\beta U\left(I,H\right)=\left(1+\beta \right)\left[v\left(I\right)+w\left(H\right)\right]$$

(2) $$E_{R}\left(u\right)=U\left(I^{+},H\right)+\beta \left[pU\left(I^{-},H^{-}\right)+\left(1-p\right)U\left(I,H\right)\right]=v\left(I^{+}\right)+w\left(H\right)+\beta \left\{p\left[v\left(I^{-}\right)+w\left(H^{-}\right)\right]+\left(1-p\right)\left[v\left(I\right)+w\left(H\right)\right]\right\}$$

The sex worker decides to engage in safe sex in period 1 if $E_{S}\left(u\right)>E_{R}\left(u\right)$, which is equivalent to:

$$\beta p\left[w\left(H\right)-w\left(H^{-}\right)\right]>v\left(I\right)-v\left(I^{+}\right)+\beta p\left[v\left(I\right)-v\left(I^{-}\right)\right]$$

To illustrate the role of risk preferences, consider the hypothetical scenario where the sex worker has a preexisting condition that affects her level of health negatively. In that scenario, both level of utilities (with or without STI) are thus reduced by a fixed quantity Δ: H'=H−Δ and $H{'}^{-}=H^{-}-\text{Δ}$.

If the sex worker is risk averse with respect to health, utility function *w* is concave, then $w\left(H'\right)-w\left(H{'}^{-}\right)=w\left(H-\text{Δ}\right)-w\left(H^{-}-\text{Δ}\right)>w\left(H\right)-w\left(H^{-}\right)$. Therefore, it could be that the sex worker chooses the risky behaviour without the preexisting condition but chooses the safe behaviour with the preexisting condition. This will be the case if:

$$\beta p\left[w\left(H'\right)-w\left(H{'}^{-}\right)\right]>v\left(I\right)-v\left(I^{+}\right)+\beta p\left[v\left(I\right)-v\left(I^{-}\right)\right]>\beta p\left[w\left(H\right)-w\left(H^{-}\right)\right]$$

By contrast, if the sex worker is risk neutral with respect to health, utility function *w* is linear, in that case there would be no difference in behaviour with or without the preexisting condition since $w\left(H'\right)-w\left(H{'}^{-}\right)=w\left(H-\text{Δ}\right)-w\left(H^{-}-\text{Δ}\right)=w\left(H\right)-w\left(H^{-}\right)$. Note that, differences in utility levels are not sufficient to characterize differences in risk preferences. For example, utility functions u(I,H)=I+H and u'(I,H)=2I+3H exhibit the same level of risk preferences (risk neutrality) despite differences in utility levels. The degree of risk aversion (with respect to both health and income) is captured by the concavity of the utility functions. Considering pre-existing shocks in health (or income) is a simple way to illustrate the role of risk aversion on individual behaviour.

Similarly, a negative income shock could have a different effect on the sex worker's behaviour depending on how risk averse she is with respect to income (the concavity of *v*).

This simple example shows why it is important to consider both risk preferences with respect to income and health in our analysis. In particular, note that the sex worker's behaviour might be affected by her risk aversion with respect to health (income), even if she is risk neutral with respect to income (health).

In order to draw predictions on the sign of the relationship between risk aversion and sexual and health behaviours and health outcomes, we refer to the distinction between self-protection and self-insurance (Erlich and Becker, 1972). Self-protection refers to any activity that reduces the probability that a bad event (e.g. the infection with STI or HIV) occurs while self-insurance refers to any activity that reduces the loss in case the bad event occurs. While the first type of activity corresponds to primary prevention, the second refers to secondary prevention. Relationships between risk aversion and self-insurance or self-protection are ambiguous. Some papers found that individuals with a greater risk aversion invest more in self-insurance (Dionne and Eeckhoudt, 1985; Grimm and Treibich, 2016). Other studies (Briys et al., 1991; Goldzahl, 2017) nuance this result, showing that if the self-insurance activity does not allow to decrease the size of the bad event, risk aversion can be negatively correlated with self-insurance. We expect that risk aversion is positively correlated with investment in self-insurance as soon as the efficiency of self-insurance is sure enough. The relationship between self-protection and risk aversion is less ambiguous and one can assume that risk aversion is positively associated with self-protection. However, the role of risk aversion may be domain specific for preventive health behaviours that have different impact on income and on health. For instance participation in community-based activities may have a direct benefit on health but no effect on income and could even be costly in terms of time. Other health behaviours, such as sex work registration, may allow both to increase earnings by accessing to a greater pool of clients and to improve health through compulsory health checks. Finally, it is assumed that sex workers who take more risks in health or sex may increase health behaviours that have important health benefits.

As for health outcomes, the relationship between HIV infection and risk aversion is ambiguous due to potential reverse causality. Low risk aversion may lead to HIV infection but being HIV infected may also affect risk aversion level. On the contrary, it is unlikely that a previous STI infection affects risk aversion given that STI can be easily cured. Therefore, the association between risk preferences and STI symptoms may either reflect the consequences of risks taken by the individual or a stronger preference for prevention. We summarise the expected relationships between risk preferences and our outcomes of interest in Table 1.

**Table 1:** Expected association between risk aversion and outcomes.

Outcomes	Type of activity	Explanation of type of activity	Expected association with risk aversion
Sexual behaviours			
Number of sex acts	Self-protection	The higher the number of sex acts, the greater	–
		the probability of being infected with STI/HIV	
Condom use	Self-protection	Condom use decreases the probability of	+
		being infected with STI/HIV	
Client at risk of HIV	Self-protection	A client at risk of HIV increases the probability	–
		of being infected with STI/HIV	
Price charged	Both	Depend on risk preferences' domain	Ambiguous
		- price charged is a proxy for risky sex (SP)	
		- price charged compensates the fear of being infected with STI (SI)	
**Health behaviours**			
Receive services from an NGO	Both	Depending on services provided	Ambiguous
		- provide free condoms (SP)	
		- testing and STI treatment (SI)	
Causerie	Both	Depend on information provided	Ambiguous
		Depend on risk preferences' domain	
Registered with authorities	Self-insurance	Decrease the loss in case of bad event through regular	Ambiguous
		medical checks that allow early treatment initiation	
		Depend on risk preferences' domain	
Had a HIV test in the last 12 months	Self-insurance	Decrease the loss in case of bad event through early treatment	Ambiguous
		Initiation	
**Health outcomes**			
STI symptoms	Both	Past risky sexual behaviours (SP)	Ambiguous
		Increased demand for medical test (SI)	
Ever had STI	Both	Past risky sexual behaviours (SP)	Ambiguous
		Increased demand for medical test (SI)	
HIV positive		Current status reflects past risky behaviours, however once infected	Ambiguous
		the individual may modified her self-declared risk preferences	

*Notes:* SP stands for self-protection and SI for self-insurance.

## 3 Experimental set up and methods

**Fig. 1:**
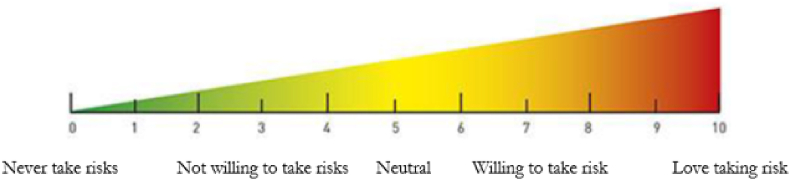
Visual scale used for self-reported risk preferences.

Risk preferences were elicited using two methods: a self-assessed measure (in four different domains) and an incentive-compatible measure. Precisely, sex workers were first asked to self-report their risk preferences (SRRP) in general, in finance, in health and with sexual behaviours using a visual scale going from 0 to 10 (see Fig. 1 Dohmen et al., 2011). Note that while the concept of risk exists and is very well understood in Senegal, there is no word for ‘risk’ in the local language (Wolof), hence it is the French term ‘risque’ that is used in Senegal and was used in the survey. We use both experimental and non-experimental methods to measure risk aversion in finance given that there is no consensus in the literature of the superiority of one method. Some have found that survey measures perform worse than experimental measure (Burks et al., 2012). In particular, a main issue often reported with survey measures is that since they are collected coterminously, it may create reverse causality issue. Others have found that survey measures perform better than experimental measures (Dohmen et al., 2011; Lonnqvist et al., 2014) especially in less numerate subjects (Charness and Viceisza, 2016; Dave et al., 2010) since the complexity of experiments leads to more noise in decision-making. Note that to measure risk aversion in health, we only use self-reported measures (preference for risk in health and sex). This is because an incentive compatible measure of risk preference raise important methodological challenges.

Following Charness and Gneezy (2010), we used a simplified version of the Gneezy and Potters (G&P) task. Participants received a fixed amount of money of CFAF 3000 (USD 5, note that at the time of the survey 1 USD = 554 CFAF) and were asked about the amount they would like to invest in a risky business and the amount they would like to keep. Specifically respondents had to choose among seven predefined amounts (0; 500; 1000; 1500; 2000; 2500 or 3000). The business was risky because it returned 2.5 times the invested amount with a probability of 50% and the invested amount was lost with a probability of 50% (see [INSERT LINK TO ONLINE FILE A] for a detailed presentation of the task). After making their choice, a random draw took place to find out if the invested amount was lost or increased. The average expected return that participants could win was set at CFAF 3300 (USD 6) but participants could win up to CFAF 7500 (USD 13.5).

Implied constant relative risk aversion (CRRA) ranges for each choice were computed by equalising the expected utility derived from the chosen option and the two adjacent options using the following formula: $U\left(x\right)=\frac{x^{\left(1-r\right)}}{\left(1-r\right)}$; *r* being the implied CRRA (Holt and Laury, 2002; Wakker, 2008).

We used the mid-point CRRA as a measure of risk aversion (Table 2). The task exerts large variability in the CRRA ranging from a CRRA greater than 2 (high risk aversion) to a CRRA close to 0 (low risk aversion). The experimental task used does not allow us to distinguish individuals who have a very low risk aversion from those who are risk neutral or risk seekers. Note that we reverted the scale of the self-reported measure so that the experimental measure is positively correlated with the self-reported ones.

**Table 2:** Task with real payments.

Amount invested	Dividend	Low pay-off	High pay-off	Expected return	Standard deviation	Implied CRRA range	Mid-point CRRA
0	0	3000	3000	3000	0	2.00 <r	2
500	1250	2500	3750	3125	625	0.67 <r< 2.00	1.33
1000	2500	2000	4500	3250	1250	0.39 <r< 0.67	0.53
1500	3750	1500	5250	3375	1875	0.67 <r< 0.27	0.33
2000	5000	1000	6000	3500	2500	0.27 <r< 0.19	0.23
2500	6250	500	6750	3625	3125	0.19 <r< 0.11	0.15
3000	7500	0	7500	3750	3750	r< 0.11	0.11

*Notes:* CRRA stands for constant relative risk aversion.

Before implementing the task, we piloted it among 50 participants and run focus groups discussions that aimed at discussing different risk tasks and assessing understanding and participants’ perception of those tasks. Note that before playing the task with real money, all participants were asked to play a training round and participants with poor understanding of the task had the possibility to participate to two additional training rounds. The results indicated that the self-reported understanding of the task was good since only eleven participants asked to play an additional training round. Those participants had the opportunity to play a third training round but none took this opportunity given that all participants declared to have understood the tasks. In order to have a more objective measure of their understanding of the task, we quizzed participants on the amount of their gain given their choice and the outcome of the random draw. We create a dummy variable that takes value of one if the respondent provided the correct amount of the gain and zero otherwise. It is worth noting that only 2.5% of individuals who did not provide the correct amount asked for an additional training round. Yet, the understanding of the task was good in the sample since 80% of all participants were able to correctly answered to the quizz (see Table 5).

## 4 Empirical specification

In order to investigate the role of risk preferences on sexual or health behaviours and health outcomes, we run the following estimations using ordinary least squares:

$$S_{i}=\beta RP_{i}+\varepsilon_{i}$$

where $S_{i}$ is one of the outcomes of interest measuring either sexual behaviours with clients, the demand for STI/HIV prevention or STI/HIV status of sex worker *i*. Note that for some of the sexual behaviours outcomes (price of sex act and riskiness of the clients), regressions include a larger number of observations since this information was collected for the last two sex acts. Standard errors were clustered at the sex worker level in those regressions. $RP_{i}$ is a measure of risk preferences of sex worker *i*. Precisely we revert the SRRP scale so that both a large SRRP and a larger CRRA measure greater risk aversion. $\varepsilon_{i}$ is an error term. Coefficients of the RP variable were standardised.

In our empirical analysis, we also consider risk preferences in different domains simultaneously. More precisely, we introduce risk preferences with respect to income ($RP_{i}^{I}$) and with respect to health or sex ($RP_{i}^{H}$) in the same regression, as follows:

$$S_{i}=\beta^{I}RP_{i}^{I}+\beta^{H}RP_{i}^{H}+\varepsilon_{i}$$

This specification allows us to investigate possible domain specificities which have been highlighted both in the literature (Barseghyan et al., 2011; Galizzi et al., 2016; Nicholson et al., 2005) and in our theoretical framework section. In our main analysis, we consider the experimental task to measure risk preferences with respect to income and self-reported risk preferences in health or sex as measures of risk preferences with respect to health. Given the existing debate on the relative performance of survey vs. experimental measures (Burks et al., 2012; Dohmen et al., 2011; Lonnqvist et al., 2014), we considered self-reported risk preferences in general and in finance in our robustness checks.

We further test the robustness of our results when controlling for a set of covariates as well as adding enumerators’ fixed effects. Covariates included altruism, preference for the present, big five personality traits, religiosity, self-control, mental health and well-being as they are found to be strongly correlated with risk aversion in the literature (Crisp and Barber, 1995; Nicholson et al., 2005; Noussair et al., 2013; Smoski et al., 2008) but also strongly associated with health outcomes (Agardh, 2012; Gafni and Torrance, 1984; Goodwin and Friedman, 2006).

## 5 Data and descriptive statistics

Participants were all female sex workers working in Dakar and the sample includes an equal proportion of registered and unregistered sex workers. Registered sex workers were recruited using medical records from four (out of the five) STI centres located in the suburb of Dakar (Rufisque, Pikine, Mbao, and Sebikotane) while unregistered sex workers were recruited through sex workers’ group leaders and NGO staff working with unregistered sex workers. Ethical clearance was obtained from the London School of Hygiene & Tropical Medicine and from the national ethics committee in Senegal. Note that although this survey took place in 2017 (Wave 2), data used in this paper comes from a cohort study initiated in 2015 (Wave 1) and 9% of sex workers who participated in Wave 1 were no longer working in sex work in Wave 2.

Characteristics of sex workers interviewed are presented in Table 3. Sex workers are on average 39 years old. Most of sex workers are divorced (66.7%) or have never been married (18.6%) and hence do not receive any financial support from their partner, which is consistent with the fact that 92% of sex workers report to have started selling sex because of financial reason. Sex workers have monthly household expenditure of CFAF 353,224 (USD 638). This corresponds to a monthly per capita expenditure of CFAF 95,640 (USD 170), which is 2.2 times higher than the level of per capita expenditure in Dakar reported in national statistics (CFAF 43,260) (ANSD, 2013). On average, sex workers reported monthly earnings from sex work of CFAF 126,551 (USD 230). Data also contain information on psychological traits, such as altruism, preference for the present, big five personality traits, religiosity and self-control as well as on mental health (self-efficacy, depression) and well-being. We measured self-efficacy among sex workers as in Ghosal et al. (2016). Note that there is some close link between self-efficacy and mental health in the psychology literature (Connolly, 1989; Murphy et al., 1987; Schönfeld et al., 2016). We measured altruism toward street children. To do so, participants were given CFAF 1000 (i.e. USD 1.81) in coins of CFAF 50 and were asked how much they would like to transfer to a recipient. The recipient in this task was a charity organisation helping street children (called *“talibés”*), a major social issue in Senegal. On average, CFAF 195 was given to the charity organisation by each participant, i.e. 20% of the amount received. Information on the preference for the present was collected in reference to finance with a choice-based method, as used in Hardisty et al. (2013), that consisted in observing behaviours of respondents given a discount factor (Frederick et al., 2002). Precisely, we asked participants if they would prefer a sum of money today or 1.5 times this amount in one week of time as commonly done and 87.7% of participants declare that they prefer receiving a lower earning immediately. Big five personality traits were measured using a 44-item inventory to which a scoring was applied in order to construct a scale for extraversion, agreeableness, conscientiousness, neuroticism and openness (John and Srivastava, 1999). Self-efficacy was constructed using a principal component analysis including a set of 11 likert scale questions aiming to capture the ability to solve problems in different domains (with police, clients, children, etc.). Finally, the patient health questionnaire PHQ-9 was used to assess whether participants suffered from depression. Based on this test, 9.0% of sex workers interviewed suffered from moderately severe or severe depression (PHQ-9 score above 14).

**Table 3:** Characteristics of sex workers.

Variables	*Obs.*	Mean	SD
Socio-demographic characteristics			
Age (in years)	*592*	38.66	9.54
Marital status:	*592*		
Never married		0.186	
Married		0.054	
Divorced		0.667	
Widowed		0.093	
Household size	*592*	8.037	5.445
Monthly earning from sex work (CFAF)^a^^,^^b^	*511*	126,551	111,413
Monthly household expenditures (CFAF)^a^	*592*	353,224	290,666
Expenses last 48 h (CFAF)^a^	*592*	11,124	7971
Not in urgent need of cash	*587*	0.317	0.466
**Psychological and personality attributes**			
Altruism (out of 1000 CFAF)^a^	*590*	195	241
Preference for the present^b^	*592*	0.877	0.329
Extraversion^c^	*592*	25.248	4.119
Agreeableness^c^	*592*	33.573	3.653
Counsciousness^c^	*592*	33.813	4.251
Openess^c^	*592*	28.848	5.072
Neuroticism^d^	*592*	21.041	4.184
Religiosity: “God protects me”	*592*		
Strongly disagree		0.000	
Disagree		0.014	
Agree		0.422	
Strongly agree		0.564	
**Mental health**			
Self-efficacy^e^	*592*	0.000	1.001
Self-control^f^	*592*	0.193	0.395
Happiness	*592*		
Not at all happy		0.015	
Not happy		0.145	
Neither happy nor not happy		0.360	
Happy		0.367	
Very happy		0.113	
Moderately severe depression^g^	*592*	0.090	0.286

*Notes:* Differences in the number of observations are due to missing information.

a 1 USD = 554 FCFA.

b Prefer 1000 CFAF now than 1500 CFAF in a week.

c Out of the 592 respondents interviewed, 62 are no longer FSWs.

d Each index is the sum of the scores of a series of questions using a scale going from 1 to 5. Questions are derived from the Big five personality traits questionnaire (44 items). Extraversion and neuroticism are based on 8 items, agreeableness and counsciousness on 9 items and openess on 10 items.

e Self-efficacy is based on a factor analysis that include 9 items that measure the ability to sort out issues with police, landlord, neighbour, clients, sudden illness, make decisions regarding child future, ability to develop skills to start a new business, ability to buy a place.

f Self-control is equal to 1 if the sex worker disagrees with “I have a good self-control”.

g This variable is equal to 1 if PHQ-9 ≥ 15 and 0 otherwise.

We analyse the effect of risk preferences on several outcomes capturing different dimensions of the demand for HIV/STI prevention (primary and secondary prevention activities) and risky sexual behaviours. Note that some outcomes could be seen as conditional or simultaneous since pairwise correlations showed some correlation between outcomes.

The first set of outcomes, risky sexual behaviours, were chosen by referring to the determinants of the probability of becoming infected with an STI and HIV, which are determined by the average number of sexual contacts given partner, the probability of transmission from single contact with an infected person and the prevalence of STI in the population (Corno and De Paula, 2019; Nelson and Williams, 2014). We include the number of sex acts with commercial clients as a proxy for the average number of sexual contacts. We proxy the probability of transmission in a single contact by condom usage and by the price charged assuming that a higher price reflects greater risk-taking during the sex act (Rao et al., 2003). We recall that those two latter variables were collected in reference to the two last commercial sex acts for active FSWs (n=1,023). Finally, we proxy the prevalence of STI of sexual partners by the perceived riskiness of the client. It may take some delay to become infected with a STI or HIV given that the probability of infection is lower than one. Note, however, that most epidemiological studies use HIV status, STI status, number of partners and sexual intercourse as outcomes as proxies for risky sexual behaviours (Dir et al., 2004; Jackson et al., 2012). It is important to note that we use the number of sexual acts as a proxy for risky sex in addition to unprotected sex. This is justified by the fact that firstly, condom rupture or condom misuse does occur and although we do not have an exact estimation of the prevalence of condom rupture in the data, we have anecdotal evidence suggesting that many sex workers incorrectly use condom. For instance, we know that many sex workers use two condoms at the same time, which leads to an increased risk of condom rupture. Secondly, one needs to bear in mind that even if the vaginal sex act is protected, sex workers rarely use a condom for oral sex and may become infected by a STI after performing this type of sex act. Given that several sexual behaviours (unprotected sex and anal sex) are considered socially unacceptable, their low proportion when asked directly (2.4% and 3.3%) prevent from using these measures. As a result, condom use was indirectly elicited using a list experiment method to overcome social desirability bias (see [INSERT LINK TO ONLINE FILE B] for details on this method).

The second set of outcomes are health related behaviours capturing the demand for STI/HIV prevention such as the affiliation to a NGO, the participation in community-based health information group meetings (*‘causerie’*), the registration status and HIV screening. Note that the current package of services offered by NGOs working to female sex workers in Senegal only focus on health services and include information on STIs and HIV, condom distribution, STI and HIV screening and STI treatment.

The last set of outcomes analysed relate to the sex worker's health and captures the presence of STI and HIV. The presence of STI is measured by the presence of various self-declared STI symptoms in the past month such as vaginal discharge, low abdominal pain and genital ulcer. The latest is a common symptom of STI frequently used as a proxy for its measure (Pandey et al., 2008). Information on HIV status was obtained from the medical records collected part of the sex work registration policy and is, as a result, only available for registered sex workers.

Descriptive statistics regarding those outcomes are reported in Table 4. On average, sex workers see about eight clients a week. 22.0% of sex workers did not use a condom during the last commercial sex act and this can be explained by the fact that sex workers evaluate that their clients have a relatively low risk of being infected with HIV (this risk was assessed to be on average 1.6 on a 10-point scale). On average, sex workers charge CFAF 16,555 (roughly USD 30) per sex act. In addition, the demand for HIV and STI prevention is high since 82.6% of sex workers were screened for HIV in the last 12 months. Finally, they are 51.4% to declare being involved in community-based HIV prevention activities.

**Table 4:** Sexual, health behaviours and health outcomes.

Variables	*Obs.*	Mean	SD
Sexual behaviours			
Number of sex act per week	*513*	8.353	8.810
Condom use during last sex act^a^	*513*	0.780	0.061 (SE)
Client is at risk of HIV^b^^,^^c^	*1023*	1.604	2.519
Price charged (CFAF)^c^	*1024*	16,555	35,009
**Health behaviours**			
Receive services from an NGO	*583*	0.249	0.432
Participate in a community-based activity ‘causerie’^d^	*588*	0.514	0.500
Registered with authorities	*512*	0.498	0.500
Had a HIV test in the last 12 months	*592*	0.826	0.379
**Health outcomes**			
Any symptom in last month (/3 symptoms)	*589*	0.144	0.352
STI symptom in last month: vaginal discharge	*591*	0.098	0.298
STI symptom in last month: lower abdominal pain	*590*	0.078	0.268
STI symptom in last month: genital ulcer	*590*	0.022	0.147
Ever had an STI	*592*	0.341	0.475
Positive HIV test among registered sex workers^e^	*173*	0.081	0.274

*Notes:* Differences in the number of observations for some variables are due to missing information.

a Condom use is measured via the list experiment method (see Appendix 2). These questions were asked only to active sex workers.

b Respondents were asked to rate the HIV riskiness of the client on a scale going from 0 to 10.

c Information on the last two sex acts with a client.

d A causerie is a group counselling usually delivered by NGOs or sex workers' leaders on the topic of HIV/AIDS.

e Information come from available medical records of registered sex workers.

Regarding respondents’ health, medical records indicate that 8.1% of registered sex workers are infected with HIV and self-reported STI prevalence assessed through symptoms is 14.4%. At the time of the survey, 34.1% of sex workers reported to have ever suffered from any STI symptoms.

## 6 Risk aversion measures

**Fig. 2:**
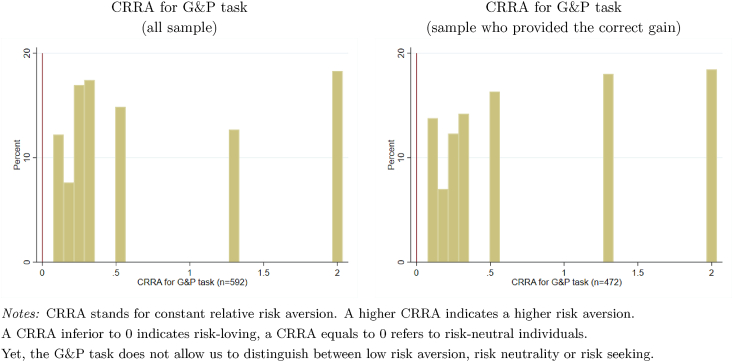
Distribution of risk aversion elicited with an incentive-compatible measure.

We first look at risk aversion elicited with the incentive-compatible measure. Fig. 2 shows that 13.56% have a CRRA close to 0.

Overall, Fig. 2 highlights large heterogeneity in risk aversion estimated using the real payment task. This is confirmed even when restricting our sample to respondents who provided the correct gain for the G&P task. If we compare risk aversion of sex workers in Senegal to risk aversion of other populations, one can note that our results confirm that risk aversion is higher in low-income countries than in Western societies. We find that on average sex workers invests 46.8% of the amount given in the G&P task, whereas overall investment levels range from 44.67% to 65.42% among student populations in Western societies (see Charness and Viceisza (2016) for a review of the G&P results in previous studies) and this difference is probably explained by the absence of formal risk-coping mechanism in Senegal. If we now compare those results to the ones reported on other populations in low-income countries, we can see that sex workers display on average greater risk preferences since the overall investment levels in the G&P task range from 23.08% to 50.03% in low-income countries. In addition, the CRRA based on the sample of Senegalese farmers in Charness and Viceisza (2016) indicates that 21% of farmers have a CRRA lower than 0.33 against 53% in our sample of sex workers, meaning that Senegalese sex workers are less risk averse than Senegalese farmers.

**Fig. 3:**
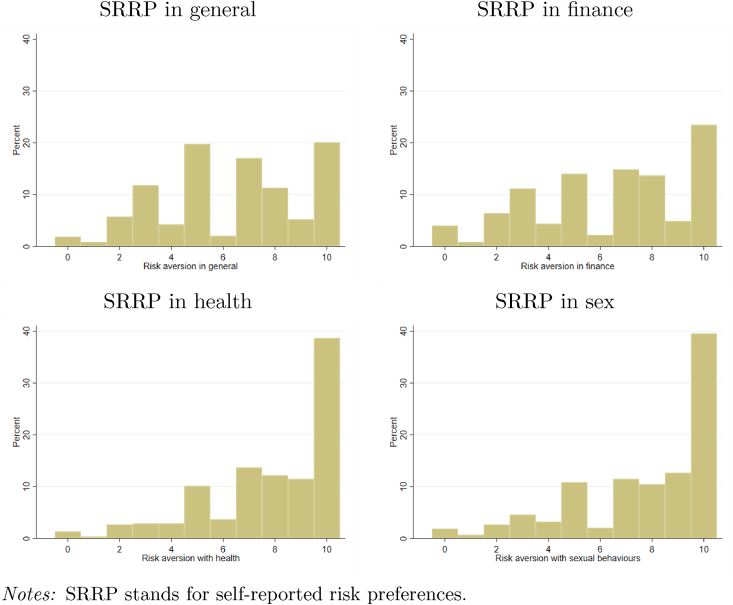
Distribution of self-reported risk preferences aversion in different domains (n=592).

Fig. 3 shows the distribution of self-reported risk preferences. The figure reveals heterogeneity in risk attitudes especially in general and for financial matters although respondents tend to be risk averse, the median for the preference for risk in general is 3. Looking at the distribution of subjective risk preferences across domains, one can note that sex workers declare taking more risks in finance than with their health or sexual behaviours and it is interesting to note that the distributions across the health and sexual domains have a similar pattern in the sense that they exhibit a large proportion of 10: 40% of sex workers are not at all willing to take risks in those domains. This greater aversion to risk in health compared to finance has also been highlighted by Galizzi et al. (2016) among Greek patients.

In order to further investigate the role of social desirability in these results, we investigated whether it was the same individuals who were declaring not being willing to take risk at all in the different domains. Table A1 in [INSERT LINK TO ONLINE FILE C] displays the pairwise correlation coefficients between binary variables that all take value one if the respondent answered that she is not willing to take any risks. We can see that there is large correlation for not reporting to take any risk in health and sex: 265 respondents (out of 592) are not willing to take risks in health or in sex, among which 74.72% (198/265) are not willing to take risk in both domains. In addition, there is large correlation for not reporting to take any risk in general and finance: 159 respondents (out of 592) are not willing to take risks in general or in finance, among which 62.26% (99/159) are not willing to take risk in both domains.

## 7 Results

Table 6 presents the effect of the different measures of risk aversion on sexual and health behaviours as well as health outcomes. Note that each reported coefficient estimate shown in Panels 1 and 1b of this table is based on a separate regression and coefficients are standardised for comparison purposes. In Panel 2 of Table 6, we introduce risk preferences in finance and in the health or sex domain in the same regression. Note that in all estimates, higher CRRA and higher SRRP means greater risk aversion. For estimates that include simultaneously risk aversion in more than one domain, we tested multicollinearity using variance inflation factors (VIF). The mean VIF was around 1 in all estimates, which does not suggest high multicollinearity.

**Table 5:** Understanding of the G&P task.

Variables	*Obs.*	Mean	SD
Correct gain	*592*	0.797	0.402
Understand task (correct probability and gain)	*592*	0.486	0.500

*Notes:* The variable ‘correct gain’ takes value 1 if the FSW was able to provide the correct gain after the random draw. The variable ‘understand task’ takes value 1 if the FSW was able to provide correct gain after the random draw and indicated that she has a 50% chance of winning before the random draw.

**Table 6:** Association between risk preferences and health, sexual behaviours and health outcomes.

	Sexual behaviours				Health behaviours				Health outcomes		
	Number of sex acts per week	Condom use	Risky client	Price (CFAF)	Affiliated NGO	Causerie	Registered	HIV test	STI symptom	Ever had a STI	HIV positive
	(1)	(2)	(3)	(4)	(5)	(6)	(7)	(8)	(9)	(10)	(11)
Expected sign	–	+	–	+/−	+/−	+/−	+/−	+/−	+/−	+/−	+/−
***Panel 1: All sample - considering each risk preferences measure seperately***											
CRRA G&P	−1.434*** (0.354)	0.099* (0.056)	−0.399*** (0.092)	−3065*** (1158)	0.037* (0.019)	0.063*** (0.021)	−0.032 (0.022)	−0.026* (0.016)	−0.029** (0.014)	−0.082*** (0.018)	0.021 (0.024)
SRRP in health	−1.736*** (0.422)	0.150** (0.059)	−0.662*** (0.097)	2671** (1250)	−0.007 (0.018)	−0.035* (0.021)	0.006 (0.022)	−0.002 (0.016)	0.010 (0.015)	0.007 (0.020)	−0.049** (0.024)
SRRP in sex	−1.554*** (0.396)	0.172*** (0.060)	−0.531*** (0.104)	1431 (1251)	−0.014 (0.019)	−0.014 (0.021)	0.053** (0.021)	0.018 (0.016)	0.013 (0.014)	0.002 (0.020)	−0.026 (0.020)
***Panel 2: All sample - considering risk preferences in finance and health or sex in the same regression***											
CRRA G&P	−1.340*** (0.338)	0.085 (0.056)	−0.362*** (0.088)	−3240*** (1220)	0.037** (0.019)	0.065*** (0.020)	−0.032 (0.022)	−0.026* (0.016)	−0.029** (0.014)	−0.083*** (0.018)	0.026 (0.024)
SRRP in health	−1.662*** (0.413)	0.149** (0.059)	−0.642*** (0.096)	2863** (1298)	−0.009 (0.018)	−0.037* (0.020)	0.007 (0.022)	−0.001 (0.016)	0.011 (0.015)	0.011 (0.020)	−0.051** (0.024)

CRRA G&P	−1.257*** (0.336)	0.081 (0.057)	−0.337*** (0.091)	−3307** (1309)	0.039** (0.019)	0.065*** (0.020)	−0.040* (0.022)	−0.028* (0.016)	−0.031** (0.014)	−0.083*** (0.018)	0.029 (0.024)
SRRP in sex	−1.399***	0.162***	−0.489***	1849	−0.018	−0.022	0.058***	0.021	0.017	0.011	−0.033
*Observations*	*513*	*513*	*1023*	*1024*	*583*	*588*	*512*	*592*	*589*	*592*	*173*
***Panel 1b: Restricting to correct gain sample***											
CRRA G&P	−1.257***	0.099	−0.452***	−3055**	0.039*	0.056**	−0.062***	−0.021	−0.001	−0.068***	0.030
	(0.377)	(0.062)	(0.103)	(1431)	(0.021)	(0.022)	(0.024)	(0.017)	(0.014)	(0.019)	(0.025)
*Observations*	*411*	*411*	*821*	*820*	*465*	*468*	*410*	*472*	*470*	*472*	*149*

Notes:***p < 0.01, **p < 0.05, *p < 0.1. Risk aversion measures are standardised. No covariate is included. Robust standard errors are reported in parentheses.

Standard errors are clustered at sex worker level for sex act level analysis (Columns (3) and (4)). Each reported coefficient estimate is based on a separate OLS regression in Panels 1 and 1b. SRRP stands for self-reported risk preferences. CRRA stands for constant relative risk aversion. Higher CRRA and SRRP mean greater risk aversion. Columns (3) and (4) refer to the two last paid sex intercourses. Column (11) comes from medical records of registered sex workers.

Differences in the number of observations in columns (5), (6) and (9) are due to missing information. Registration status information (Column (7)) is available for active FSWs only. In column (2), the reported coefficients refer to the interaction term $RP_{i}\times T_{i}$, see Appendix 2.

### 7.1 Sexual behaviours

The main result we can see from Table 6 is that there is an association between risk aversion measures and sexual behaviours. More precisely, it is interesting to note that the results obtained with the experimental measure of risk aversion in finance and the self-reported measure of risk preferences in health and sex are consistent for three out of the four sexual behaviours considered (number of sex acts, condom use, risky client). For these outcomes, the coefficient of the two types of measures are of a similar magnitude.

More precisely, risk averse sex workers are found to have a lower average number of sex acts with clients: an increase in risk aversion in finance by one standard deviation decreases the average number of sex act with clients per week by 1.4. Similar results are found when considering self-reported risk preferences in health or in sex. Furthermore, there is a clear pattern showing that risk averse sex workers are less likely to take risk during sex acts. Indeed, there is a strong association between self-reported risk preferences and condom use. Precisely, an increase in one standard deviation in risk aversion in finance increases the likelihood to use condom by 9.9 points and an increase in one standard deviation in self-reported risk preferences in health and sex increases condom use by 15.0 and 17.2 percentage points respectively. Results also indicate that risk preferences are correlated with characteristics of the clients since sex workers who are more risk averse are less likely to engage in sex with a risky client, this whatever the risk aversion domain considered. For example, an increase of one standard deviation in risk aversion in finance decreases the perceived riskiness of the client by 0.4 points (on a 10 point scale). The above results are in line with the expected relationships presented in Table 1.

When investigating the relationship between risk preferences and price of a sexual intercourse, and assuming that price captures the risk taken during sex act, we can distinguish two opposite effects depending on the domain examined. On the one hand, the results confirm that sex workers who are more risk averse with finance charge a lower price of CFAF 3065 (or USD 5.5) on average for a one standard deviation increase in risk aversion. On the other hand, an increase in one standard deviation in risk aversion in health increases the price by CFAF 2671 (or USD 4.8), which can be interpreted as a financial compensation for the additional health risks taken. These effects are confirmed when both risk aversion measures are introduced simultaneously in the same regression (cf. Panel 2 of Table 6).

### 7.2 Health behaviours

We now turn to the analysis of health behaviours (e.g. affiliation to an NGO, use of community-based health services, registration, HIV test).

We find that risk aversion in finance is positively associated with the affiliation to an NGO and the participation in community-based health activities. It is found that an increase in one standard deviation in risk aversion in finance is associated with an increase in the likelihood to participate in community-based health activities by 3.7 points and increases the likelihood of participating in a ‘causerie’ by 6.3 percentage points (cf. Panel 1 in Table 6). These results reflect a lower demand for prevention for sex worker who have lower risk aversion in finance. On the contrary, we detect a negative relationship between risk preferences in health and the likelihood of participating in a ‘causerie’, which may reflect a higher demand for prevention for sex workers who take more risks with their health. These two opposite effects remain statistically significant when introducing both risk preference measures in the same regression (cf. Panel 2). As for the decision to register, we also detect different results depending on the risk aversion domain. Sex workers who are more willing to take risks with finance are more likely to register, which capture the fact that registration offers the possibility to increase earnings by accessing a larger pool of clients. Conversely, those who have higher level of risk aversion in sex are more likely to register. This result may be related to the fact that registration has been found to increase stigma and deteriorates self-image (Ito et al., 2018) and that sex workers who have greater risk preferences for sex may hence be more reluctant to register to preserve their self-image. Finally, we also find that an increase in one standard deviation in risk aversion in finance is associated with a decrease in the likelihood of testing for HIV by 2.6 points, confirming previous negative relationship between risk aversion in finance and the demand for screening (Goldzahl, 2017; Picone et al., 2004). However, self-reported risk preference measures in health or in sex do not appear to be significantly associated with HIV screening.

### 7.3 Health outcomes

Regarding health outcomes, based on the experimental measure of risk aversion, we see that an increase in one standard deviation in risk aversion leads to a decrease in the likelihood of having any STI symptoms by 2.9 percentage points and to a decrease in the likelihood of having had an STI by 8.2 percentage points. No significant association is detected between STI symptoms and self-reported risk preferences in health or in sex. Lastly, while there is no statistically significant relationship between risk aversion in finance and HIV status, a decrease in self-reported risk aversion in health by one standard deviation translates into a lower probability of infection of 4.9 percentage points.

### 7.4 Robustness checks

We show that our results are stable when we restrict the sample to the participants who understood the game. The only difference when doing so is that we now find that the negative relationship between risk aversion in finance and registration is stronger (see Panel 1b, Table 6). When considering self-reported risk preferences in general or in finance instead of the experimental task (Table A2 in the [INSERT LINK TO ONLINE FILE D]), similar results are found when investigating the association between risk preferences and sexual behaviours. However, no association is detected between SRRP in finance and health behaviours. Finally, domain specificities are detected when we investigate the relationship between risk aversion and the likelihood of suffering from STI symptoms. Women who are more willing to take risk in finance (in health) are more (less) likely to have ever had a STI. These results are in line with the mechanisms highlighted with respect to the price premium.

In order to investigate the relative size of the role of risk preferences, the outcomes of interest are regressed on a set of standardised socio-demographic factors. This standardisation of the explanatory variables allows us to compare the magnitude of the estimated coefficients. Table 7 shows that based on the full sample, risk preference is an important predictor of behaviours compared to other psychological and socio-demographic factors. The coefficient of risk aversion is similar than the ones of other determinants of risky behaviours. For instance, an increase of one standard deviation in risk aversion in finance increases the likelihood of having protected sex by 12.2 percentage points, while one standard deviation in income increases condom use by 6.9 points. Thus, risk aversion is found to be one of the individual characteristics influencing sexual behaviours (e.g. number of sex acts with clients, risky clients, price) and STI prevalence.

**Table 7:** Association between risk preferences and health, sexual behaviours and health outcomes - including covariates.

	Sexual behaviours				Health behaviours				Health outcomes		
	Number of sex acts per week	Condom use	Risky client	Price (CFAF)	Affiliated NGO	Causerie	Registered	HIV test	STI symptom	Ever had a STI	HIV positive
	(1)	(2)	(3)	(4)	(5)	(6)	(7)	(8)	(9)	(10)	(11)
Expected sign	–	+	–	+/−	+/−	+/−	+/−	+/−	+/−	+/−	+/−
CRRA G&P	−1.058*** (0.348)	0.122** (0.055)	−0.246*** (0.090)	−2560*** (913)	0.037** (0.019)	0.055*** (0.021)	−0.010 (0.022)	−0.022 (0.016)	−0.028** (0.014)	−0.077*** (0.019)	0.024 (0.025)
Age (in years)	−1.353*** (0.375)	0.003 (0.034)	0.043 (0.105)	−6.655 (882)	0.018 (0.020)	0.051** (0.022)	−0.001 (0.023)	0.024 (0.020)	−0.035** (0.015)	−0.058*** (0.020)	0.039 (0.028)
Expenditures last 48 h (CFAF, log)	0.611* (0.339)	0.069** (0.031)	−0.151* (0.080)	878.721 (1524)	0.002 (0.017)	0.016 (0.022)	0.124*** (0.026)	0.022 (0.018)	0.013 (0.012)	0.013 (0.016)	−0.012 (0.026)
Household size	−0.098 (0.397)	0.012 (0.038)	−0.034 (0.094)	−3455** (1669)	0.029 (0.020)	0.021 (0.021)	−0.079*** (0.022)	−0.020 (0.018)	0.006 (0.015)	0.008 (0.018)	0.038* (0.021)
Never married	0.859* (0.450)	−0.040 (0.032)	0.140 (0.095)	1906 (2328)	−0.018 (0.018)	−0.040* (0.021)	0.017 (0.022)	−0.008 (0.018)	0.007 (0.016)	0.031 (0.020)	−0.001 (0.020)
Altruism (out of 1000 CFAF)	0.034 (0.385)	0.019 (0.030)	0.093 (0.093)	678 (1107)	0.002 (0.018)	0.040* (0.021)	0.109*** (0.019)	0.021 (0.014)	−0.037*** (0.012)	−0.005 (0.019)	0.011 (0.020)
Preference for present	0.100 (0.423)	−0.043 (0.030)	0.064 (0.096)	890 (862)	0.001 (0.018)	−0.016 (0.020)	−0.021 (0.022)	−0.011 (0.015)	−0.025 (0.016)	−0.010 (0.018)	0.026* (0.014)
Extraversion	−0.556 (0.405)	0.013 (0.034)	−0.127 (0.104)	−1123 (1569)	0.028 (0.020)	0.037 (0.023)	−0.026 (0.026)	−0.009 (0.019)	0.017 (0.015)	0.028 (0.020)	−0.029 (0.025)
Agreeableness	0.004 (0.462)	−0.095*** (0.035)	−0.079 (0.113)	5220 (3322)	−0.018 (0.021)	0.015 (0.024)	−0.006 (0.026)	0.002 (0.019)	0.021 (0.016)	0.035 (0.021)	0.000 (0.023)
Conscientiousness	0.248 (0.445)	0.028 (0.033)	−0.011 (0.100)	−3,5378* (2008)	−0.031* (0.019)	0.030 (0.023)	0.001 (0.025)	0.029 (0.018)	−0.013 (0.017)	−0.012 (0.021)	−0.050* (0.026)
Openness	1.249** (0.494)	−0.076** (0.034)	0.484*** (0.108)	5688* (3394)	−0.053** (0.021)	−0.054** (0.024)	0.031 (0.025)	−0.003 (0.018)	0.000 (0.014)	−0.023 (0.021)	−0.031 (0.028)
Neuroticism	0.883** (0.444)	0.046 (0.032)	0.021 (0.101)	−2512** (977)	−0.050** (0.020)	−0.002 (0.022)	0.003 (0.025)	0.002 (0.017)	0.016 (0.013)	0.039** (0.019)	0.033 (0.024)
Reliogisity	0.419 (0.444)	0.014 (0.034)	−0.057 (0.109)	185 (927)	−0.005 (0.020)	−0.026 (0.023)	0.064*** (0.024)	0.006 (0.017)	−0.090*** (0.017)	−0.131*** (0.021)	0.011 (0.027)
Self-efficacy	0.660 (0.566)	0.042 (0.034)	0.064 (0.105)	687 (830)	0.016 (0.020)	0.022 (0.023)	0.025 (0.025)	0.006 (0.017)	−0.015 (0.016)	0.019 (0.021)	0.041* (0.023)
Self-control	−0.331 (0.385)	0.003 (0.034)	0.305*** (0.095)	606 (850)	0.003 (0.018)	−0.032 (0.022)	−0.021 (0.023)	0.014 (0.018))	0.022 (0.016)	0.018 (0.019)	0.018 (0.023
Happiness	−1.167** (0.459)	0.086*** (0.032)	−0.411*** (0.112)	2441* (1443)	0.008 (0.019)	0.044** (0.022)	−0.022 (0.023)	−0.002 (0.017)	−0.020 (0.015)	−0.063*** (0.019)	−0.014 (0.019)
Moderately severe depression	−0.210 (0.238)	−0.012 (0.028)	−0.422*** (0.071)	−1577** (639)	0.090***(0.021)	0.051** (0.020)	−0.010 (0.021)	−0.001 (0.016)	−0.015(0.015)	−0.048*** (0.018)	0.014 (0.022)
*Observations*	*511*	*511*	*1023*	*1024*	*581*	*586*	*510*	*590*	*587*	*590*	*173*
R-squared	0.130	0.296	0.134	0.082	0.094	0.081	0.135	0.027	0.101	0.157	0.137

*Notes:* Explanatory variables are standardised. Robust standard errors are reported in parentheses. Standard errors are clustered at sex worker level for sex act level analysis (Columns (3) and (4)). ***p < 0.01, **p < 0.05, *p < 0.1. CRRA stands for constant relative risk aversion. Higher CRRA means greater risk aversion. In column (2), the reported coefficients refer to the interaction term $RP_{i}\times T_{i}$, see Appendix 2. Estimates include covariates presented in Table 3.

As a robustness check, we also investigate whether FSWs' and enumerators' characteristics were likely to influence the relationship between risk preferences and behaviours. We find that the associations between risk preferences, obtained with the experimental measure, and sexual, health behaviours and health outcomes remain similar when controlling for FSWs' and enumerators’ characteristics (see Table 8). Finally, we excluded the individuals who were not willing to take any risks with health and sex on the one hand and those who were not willing to take any risks in general and in finance on the other hand given the strong correlation reported in the above tables for those two sets of variables. We can see that doing so led to minor changes in the results (cf. Table A3 [INSERT LINK TO ONLINE FILE E]).

**Table 8:** Association between G&P risk preferences and health, sexual behaviours and health outcomes - robustness checks.

	Sexual behaviours				Health behaviours				Health outcomes		
	Number of sex acts per week	Condom use	Risky client	Price (CFAF)	Affiliated NGO	Causerie	Registered	HIV test	STI symptom	Ever had a STI	HIV positive
	(1)	(2)	(3)	(4)	(5)	(6)	(7)	(8)	(9)	(10)	(11)
Expected sign	–	+	–	+/−	+/−	+/−	+/−	+/−	+/−	+/−	+/−
*All sample*											
CRRA G&P	−1.434*** (0.354)	0.099* (0.056)	−0.399*** (0.092)	−3065*** (1158)	0.037* (0.019)	0.063*** (0.021)	−0.032 (0.022)	−0.026* (0.016)	−0.029** (0.014)	−0.082*** (0.018)	0.021 (0.024)
*Controlling for sex worker characteristics*^a^											
CRRA G&P	−1.058*** (0.348)	0.122** (0.055)	−0.246*** (0.090)	−2560*** (913)	0.037** (0.019)	0.055*** (0.021)	−0.010 (0.022)	−0.022 (0.016)	−0.028** (0.014)	−0.077*** (0.019)	0.024 (0.025)
*Including enumerator CRRA G&P*											
CRRA G&P	−1.351*** (0.341)	0.086 (0.054)	−0.273***(0.094)	−2717** (1073)	0.023 (0.020)	0.034 (0.021)	−0.041* (0.023)	−0.021 (0.016)	−0.018 (0.014)	−0.048** (0.019)	0.025 (0.025)
*Including enumerator characteristics*^b^											
CRRA G&P	−1.408*** (0.361)	0.101* (0.054)	−0.208*** (0.080)	−2168** (961)	0.004 (0.018)	−0.000 (0.021)	−0.056** (0.023)	−0.030* (0.016)	−0.013 (0.015)	−0.048** (0.019)	0.032 (0.027)
*Observations*	*513*	*513*	*1023*	*1024*	*583*	*588*	*512*	*592*	*589*	*592*	*173*

*Notes:* Risk aversion measures are standardised. Robust standard errors are reported in parentheses. Standard errors are clustered at sex worker level for sex act level analysis (Columns (3) and (4)). Each reported coefficient estimate is based on a separate OLS regression. ***p < 0.01, **p < 0.05, *p < 0.1.SRRP stands for self-reported risk preferences. CRRA stands for constant relative risk aversion. Higher CRRA and lower SRRP mean greater risk aversion. Columns (3) and (4) refer to the two last paid sex intercourses.

a Sex worker characteristics are age, last days expenditures, household size, marital status, altruism, preference for present, big five personality trait, religiosity, self-efficacy, self-control, happiness and depression index (cf. Table 7).

b Enumerator characteristics include CRRA G&P, age, marital status, children, experience in surveying sex workers and years of experience in surveys.In column (2), the reported coefficients refer to the interaction term $RP_{i}\times T_{i}$, see Appendix 2.

## 8 Discussion

We measured risk preferences of female sex workers in Senegal in order to investigate the role of risk preferences in HIV/AIDS transmission. Our main result suggests that risk preferences are an important predictor of sexual behaviours and are correlated with health behaviours and with STI infection. Our results reinforce the importance of targeting high-risk female sex workers who seem to exhibit greater risk preferences than the rest of the population of female sex workers. This finding provides another explanation for the difficulty to limit the spread of the AIDS epidemic, especially in countries like Senegal, where the epidemic is concentrated among this population.

The association between the incentivised experimental risk aversion measure and sexual behaviours may lie in the fact that the decision to engage in risky sex acts involves significant personal health risks that are financially rewarded. This specific setting may explain why risk aversion measures in the financial domain is a relevant predictor of sexual behaviours of sex workers and why we detect domain specificities when considering risk preferences either with respect to finance or with respect to health. Surprisingly, self-reported risk preferences in health and in sex were not more correlated with health, sexual behaviours and health outcomes than self-reported risk preferences in finance, which might be due to social desirability bias that led to poorer variability and measurement error in those variables.

There is an emerging literature indicating that experimental measures perform poorly outside the student population found in university labs (Chuang and Schechter, 2015). In low-income countries, low level of education, poor numeracy skills and economic scarcity faced by participants can lead to bias in decision-making (Shah et al., 2012). We test the robustness of our results by taking into account the understanding of the task by participants. While most results remain similar, we detect some differences. As a result, it is crucial to design simple tasks and to assess the understanding of the task by participants. We encourage researchers who would like to use experimental measures of risk preferences in low-income countries to run focus group discussions before and after playing incentivised tasks in order to ensure that the design of the task is contextually-relevant and easy to understand.

Our results were based on two different measures of risk aversion (experimental and self-reports). We showed that the two measures led to similar associations between risk aversion and sexual behaviours. However, we show that the experimental measure was a better predictor of health behaviours than the self-reported measures. Overall, this finding suggests that when the budget allows it, risk preferences should be measured using incentivised experiments. However, given the simplicity and low cost associated with the collection of self-reported measures, the fact that there were some correlations between these measures and sexual behaviours, health behaviours and health outcomes and the importance of domain specificities highlighted in this paper when considering certain sexual or health behaviours, such risk preference measures should always be introduced in health and sexual surveys.

While this study makes new contributions, it has several limitations. The first one is that while we show that domain specific risk preferences certainly apprehend different mechanisms at play, within a same domain, different measurement techniques (here G&P task and SRRP) may capture different aspect of individual risk preferences. Secondly, we cannot exclude the fact that hypothetical measures could lead to lower levels of risk aversion than incentive compatible measures. Thirdly, the large correlation observed between self-reported risk preferences may partly be explained by an anchoring effect given that these measures were derived successively. Fourthly, our data is cross-sectional and hence it does not allow to test whether risk preferences are a stable psychological trait among the population of sex workers. Lastly, the domain complementarity highlighted may be explained by the fact that risky health behaviours of sex workers get a direct financial reward and might not be generalisable to sexual behaviours of other groups.

Our results have some policy implications. In the context of Senegal, the use of a visual scale measuring SRRP could be introduced in the enrolment form of the registration policy, which would allow health providers to identify sex workers who are at a greater risk of HIV/AIDS. We showed that the SRRP was correlated with several outcomes and such addition to the registration form would be inexpensive. Moreover, our results provide some justification regarding the effectiveness of the use of lottery based financial incentives in order to reduce STIs. In Lesotho, Björkman Nyqvist et al. (2018) found that a public lottery conditional on negative test results for STIs leads to a reduction of 21.4% in HIV incidence over a two-year period, consistent with the assumption that lotteries are particularly effective to change behaviours of individuals exhibiting larger risk preferences. Other successful interventions using lottery based financial incentives to reduce the spread of infectious diseases includes the tuberculosis-screening campaign in Scotland (https://www.theglasgowstory.com/image/?inum=TGSE00889) and the HIV screening lottery launched in 2011, which enters anyone who gets an HIV test into a drawing for cash prizes up to nearly USD 6000 (https://www.iol.co.za/news/south-africa/western-cape/zilles-hiv-campaign-gains-momentum-1189372/#.UOCcQoWYvrM). The effectiveness of lottery-based incentives depend on context and in Muslim countries like Senegal there are strong reasons to believe that such interventions would be ineffective given that gambling is not socially acceptable. This element has been confirmed by recent trials that showed that lottery-based rewards are less effective than fixed-amount financial incentives to change HIV related behaviours in some contexts like in Kenya (Thirumurthy et al., 1999) and Malawi (Choko et al., 2017). Overall, additional research is required in order to identify effective interventions in populations with different levels of risk preferences.

## CRediT authorship contribution statement

**Aurélia Lépine:** Conceptualization, Data curation, Formal analysis, Writing - original draft, Writing - review & editing. **Carole Treibich:** Conceptualization, Data curation, Formal analysis, Writing - original draft, Writing - review & editing.
