# Supplementary material for: Risk aversion and HIV/AIDS: Evidence from Senegalese female sex workers
Source: Soc Sci Med. 2020 Jul;256:113020. doi: 10.1016/j.socscimed.2020.113020 (PMC7306163; doi:10.1016/j.socscimed.2020.113020)
Supplement: Supplementary file 1 — Multimedia component 1 [file mmc1.pdf]

Appendices

Appendix 1 - Experimental instructions

READ: "In this task, you start with an amount of CFAF 3,000. You have to decide how many francs you want to invest in a small business (between 0 and 3,000 CFAF) where you have one chance out of two to lose the amount invested (if you draw the white ball with a black cross) and one chance out of two to win 2.5 times this amount (if you draw the white ball).

For instance, let's imagine that you decide to put CFAF 1,000 in this business. Your earnings will amount to CFAF 2,000 if you lose — if you draw the white ball with the black cross $(3000 - 1000)$. Your earnings will amount to CFAF 4,500 if you win — if you draw the white ball $(2,000 + 2,500)$. In other words, you are sure to keep the amount of money that you won't invest in the small business but you may increase your earnings if you invest in this business.

The table below presents the possible gains.

Investment	**Low earning** If white ball with a black cross	**High earning** If white ball
3,000	0	7,500
2,500	500	6,750
2,000	1,000	6,000
1,500	1,500	5,250
1,000	2,000	4,500
500	2,500	3,750
0	3,000	3,000

All earnings are in in CFAF.

We will first start by doing a training round in order to make sure that you well understood this task. You will then randomly draw a ball among four balls places in a black bag. Two of these balls are white with a black cross and two of them are just white. It is thus as likely that you draw a white ball with a cross or a white ball.

QUESTION: *How much money are you willing to invest in this task?* INTERVIEWER: Once the respondent made her choice, please ask her what are the amounts she will earn with this choice. This in order to be sure she well understood the decision she took.

QUESTION: *On a scale going from 0 to 100, in your view, what is the probability that you draw a white ball?*
For those who do not say 50, INTERVIEWER: Remind the participant that she is going to draw a ball in the bag and that the probability of winning the amount does not depend on her

expertise in running a business but that there is a one in two chance that she wins and a chance out of two that she loses.

READ: “You will now randomly draw a ball in this black bag in order to know what is the amount you would have won if this task is drawn and if this was not a training round. You have drawn out: *White ball with a black cross on it/ White ball*

INTERVIEWER : Ask the respondent which amount she would have earned if this was the real task and report the amount.

We will now proceed with the real task. QUESTION:*How much money are you willing to invest in this task?*
QUESTION: *On a scale going from 0 to 100, in your view, what is the probability that you draw a white ball?*

READ: “You will now randomly draw a ball in this black bag in order to know what is the amount you would have won if this task is. You have drawn out: *White ball with a black cross on it/ White ball*”
INTERVIEWER: Ask the respondent which amount she thinks she has earned and report the amount.
INTERVIEWER: Note and announce how much the respondent won.
INTERVIEWER: Ask the supervisor to come and proceed with the payment.

Appendix 2 - List experiment

The principle of the list experiment is to allocate respondents randomly to two different groups: a "control" and a "treatment" group. Individuals allocated to the "control" group are presented with a number of non-sensitive statements. They are not asked to say whether they agree on each of the statements but only with how many of them they agree on. The same statements are presented to the "treated" group; the difference is that a sensitive statement is added to the series of non-sensitive statements. Assuming that the two groups have a similar opinion of the non-sensitive statements, one can deduce the share of individuals in the "treated" group who agreed with the sensitive item by comparing the average number of agreed statements in each group (see Glynn, 2013; Holbrook and Krosnick, 2010; Kuklinski et al., 1997).

In the survey the "control" group was presented with the following question:

I [the interviewer] will read three statements. I will then ask you with how many of these statements you agree on. You should not tell me which specific statement you agree on but the number of statements you agree on. I will give you three marbles and you have to hold them in your right hand. Keep both of your hands on your back side. For each of the statements, if you agree on it, please transfer one marble from your right hand to your left hand behind you. If you do not agree on it, please do not transfer any marble. At the end, I would like to know the total number of statements you agreed on. This number should correspond to the number of marbles you have in your left hand. I will now read the statements.

1. *It is safer to bring a client home than going in a hotel.*
2. *I prefer that the client pays me before the intercourse.*
3. *Monday is the day I have the greatest number of clients.*

Participants in the "treatment" group were presented the same statements plus the sensitive item that relates to condom use.

4. *I used a condom during my last sexual intercourse with a client.*

We can investigate the relation between condom use and respondents' risk preferences using a simple linear regression with interaction terms:

$$Y_i = \beta T_i + \gamma RP_i + \alpha RP_i \times T_i + \varepsilon_i$$

where Y_i is the number of statements the respondent agreed with. T_i takes value 1 if the individual was in the treatment group. RP_i is a characteristics of individual i that may be correlated with condom use. The p-value of the coefficient α indicates if the condom use depends on the individual's risk preferences.

Appendix 3 - Pairwise correlations for FSWs who report not being willing to take any risk

Table A1: Pairwise correlations for FSWs who report not being willing to take any risk

	**Take no risk in SRRP**			
	in general	in finance	in health	in sex
No risk in SRRP in general	1.000			
No risk in SRRP in finance	0.707	1.000		
No risk in SRRP in health	0.467	0.550	1.000	
No risk in SRRP in sex	0.474	0.571	0.763	1.000
Invest no money in the game	0.073	0.089	0.086	0.135

Notes: SRRP stands for self-reported risk preferences.

Appendix 4: Association between self-reported risk preferences in different domains and sexual, health behaviours and health outcomes

Table A2: Association between risk preferences and health, sexual behaviours and health outcomes

	**Sexual behaviours**				**Health behaviours**				**Health outcomes**		
	Number of sex acts per week	Condom use	Risky client	Price (CFAF)	Affiliated NGO	Causerie	Registered	HIV test	STI symptom	Ever had a STI	HIV positive
	(1)	(2)	(3)	(4)	(5)	(6)	(7)	(8)	(9)	(10)	(11)
Expected sign	-	+	-	+/-	+/-	+/-	+/-	+/-	+/-	+/-	+/-
Panel 1: All sample - considering each risk preferences measure seperately											
SRRP in finance	-1.311***	0.162***	-0.161*	-1,751	-0.025	-0.024	0.012	0.015	-0.019	-0.053***	-0.043*
	(0.355)	(0.059)	(0.094)	(2,503)	(0.019)	(0.021)	(0.022)	(0.015)	(0.015)	(0.019)	(0.022)
SRRP in general	-1.265***	0.147**	-0.096	-2,142	-0.003	-0.009	0.014	0.020	-0.029**	-0.068***	-0.028
	(0.386)	(0.059)	(0.099)	(2,805)	(0.019)	(0.021)	(0.022)	(0.015)	(0.015)	(0.019)	(0.019)
SRRP in health	-1.736***	0.150**	-0.662***	2,671**	-0.007	-0.035*	0.006	-0.002	0.010	0.007	-0.049**
	(0.422)	(0.059)	(0.097)	(1,250)	(0.018)	(0.021)	(0.022)	(0.016)	(0.015)	(0.020)	(0.024)
SRRP in sex	-1.554***	0.172***	-0.531***	1,431	-0.014	-0.014	0.053**	0.018	0.013	0.002	-0.026
	(0.396)	(0.060)	(0.104)	(1,251)	(0.019)	(0.021)	(0.021)	(0.016)	(0.014)	(0.020)	(0.020)
Panel 2: All sample - considering risk preferences in finance and health or sex in the same regression											
SRRP in finance	-0.636*	0.108	0.190*	-3,828	-0.028	-0.010	0.012	0.021	-0.032*	-0.075***	-0.024
	(0.365)	(0.068)	(0.098)	(3,699)	(0.021)	(0.024)	(0.025)	(0.017)	(0.018)	(0.022)	(0.024)
SRRP in health	-1.441***	0.105	-0.750***	4,445	0.007	-0.030	-0.000	-0.012	0.026	0.044*	-0.035
	(0.447)	(0.070)	(0.107)	(2,796)	(0.021)	(0.024)	(0.025)	(0.018)	(0.017)	(0.023)	(0.027)
SRRP in finance	-0.742**	0.103	0.113	-3,093	-0.024	-0.023	-0.016	0.008	-0.034*	-0.072***	-0.042
	(0.357)	(0.067)	(0.100)	(3,708)	(0.021)	(0.024)	(0.024)	(0.017)	(0.018)	(0.023)	(0.026)
SRRP in sex	-1.211***	0.124*	-0.583***	2,860	-0.002	-0.003	0.060**	0.014	0.030*	0.038*	-0.003
	(0.408)	(0.068)	(0.115)	(2,790)	(0.021)	(0.024)	(0.024)	(0.018)	(0.017)	(0.023)	(0.024)
Observations	*513*	*513*	*1,023*	*1,024*	*583*	*588*	*512*	*592*	*589*	*592*	*173*

Notes: *** $p<0.01$, ** $p<0.05$, * $p<0.1$. Risk aversion measures are standardised. No covariate is included. Robust standard errors are reported in parentheses. Standard errors are clustered at sex worker level for sex act level analysis (Columns (3) and (4)). Each reported coefficient estimate is based on a seperate OLS regression in Panels 1 and 1b. SRRP stands for self-reported risk preferences. Higher SRRP mean greater risk aversion. Columns (3) and (4) refer to the two last paid sex intercourses. Column (11) comes from medical records of registered sex workers. Differences in the number of observations in columns (5), (6) and (9) are due to missing information. Registration status information (Column (7)) is available for active FSWs only. In column (2), the reported coefficients refer to the interaction term $RP_i \times T_i$, see Appendix 2.

Appendix 5: Estimations excluding FSWs who report not being willing to take any risk

Table A3: Association between risk preferences and sexual, health behaviours and health outcomes - excluding FSWs who report not being willing to take any risks

	**Sexual behaviours**				**Health behaviours**				**Health outcomes**		
	Number of sex acts per week (1)	Condom use (2)	Risky client (3)	Price (CFAF) (4)	Affiliated NGO (5)	Causerie (6)	Registered (7)	HIV test (8)	STI symptom (9)	Ever had a STI (10)	HIV positive (11)
Expected sign	-	+	-	+/-	+/-	+/-	+/-	+/-	+/-	+/-	+/-
All sample											
SRRP in general	-1.265***	0.147**	-0.096	-2,142	-0.003	-0.009	0.014	0.020	-0.029**	-0.068***	-0.028
	(0.386)	(0.059)	(0.099)	(2,805)	(0.019)	(0.021)	(0.022)	(0.015)	(0.015)	(0.019)	(0.019)
SRRP in finance	-1.311***	0.162***	-0.161*	-1,751	-0.025	-0.024	0.012	0.015	-0.019	-0.053***	-0.043*
	(0.355)	(0.059)	(0.094)	(2,503)	(0.019)	(0.021)	(0.022)	(0.015)	(0.015)	(0.019)	(0.022)
Observations	*513*	*513*	*1,023*	*1,024*	*583*	*588*	*512*	*592*	*589*	*592*	*173*
Excluding those who answered "not willing at all to take risk in general and in finance"											
SRRP in general	-1.345***	0.146*	0.434***	-3,100	-0.037*	-0.072***	0.019	0.004	-0.032*	-0.056**	-0.021
	(0.501)	(0.079)	(0.140)	(3,796)	(0.022)	(0.026)	(0.028)	(0.019)	(0.018)	(0.025)	(0.024)
SRRP in finance	-1.345***	0.162**	0.233*	-2,265	-0.063***	-0.083***	0.015	-0.000	-0.016	-0.035	-0.044
	(0.417)	(0.072)	(0.124)	(3,067)	(.021)	(0.025)	(0.026)	(0.018)	(0.019)	(0.024)	(0.028)
Observations	*432*	*432*	*861*	*862*	*485*	*490*	*431*	*493*	*480*	*493*	*139*
All sample											
SRRP in health	-1.736***	0.150**	-0.662***	2,671**	-0.007	-0.035*	0.006	-0.002	0.010	0.007	-0.049**
	(0.422)	(0.059)	(0.097)	(1,250)	(0.018)	(0.021)	(0.022)	(0.016)	(0.015)	(0.020)	(0.024)
SRRP in sex	-1.554***	0.172***	-0.531***	1,431	-0.014	-0.014	0.053**	0.018	0.013	0.002	-0.026
	(0.396)	(0.060)	(0.104)	(1,251)	(0.019)	(0.021)	(0.021)	(0.016)	(0.014)	(0.020)	(0.020)
Observations	*513*	*513*	*1,023*	*1,024*	*583*	*588*	*512*	*592*	*589*	*592*	*173*
Excluding those who answered "not willing at all to take risk in health and in sex"											
SRRP in health	-1.609***	0.140*	-0.093	2,768**	-0.043*	-0.090*	-0.007	-0.019	-0.007	-0.012	-0.050
	(0.542)	(0.083)	(0.145)	(1,397)	(0.023)	(0.027)	(0.028)	(0.021)	(0.018)	(0.026)	(0.031)
SRRP in sex	-1.302**	0.174**	0.098	735	-0.052**	-0.054**	0.069***	0.014	-0.002	-0.020	-0.011
	(0.506)	(0.082)	(0.149)	(1,321)	(0.023)	(0.027)	(0.027)	(0.020)	(0.017)	(0.024)	(0.025)
Observations	*346*	*346*	*689*	*690*	*387*	*391*	*345*	*394*	*392*	*394*	*118*

Notes: Risk aversion measures are standardised. Robust standard errors are reported in parentheses.
Standard errors are clustered at sex worker level for sex act level analysis (Columns (3) and (4)). Each reported coefficient estimate is based on a seperate OLS regression. *** $p<0.01$, ** $p<0.05$, * $p<0.1$. SRRP stands for self-reported risk preferences. CRRA stands for constant relative risk aversion. Higher CRRA and lower SRRP mean greater risk aversion. Columns (3) and (4) refer to the two last paid sex intercourses.

References

Adam N. Glynn. What can we learn with statistical truth serum? design and analysis of the list experiment. *Public Opinion Quarterly*, 77(S1):159–72, 2013.

Allyson L. Holbrook and Jon A. Krosnick. Social desirability bias in voter turnout reports: Tests using the item count technique. *Public Opinion Quarterly*, 74(1):37–67, 2010.

J.H. Kuklinski, M.D. Cobb, and M. Gilens. Racial attitudes and the 'new south. *The Journal of Politics*, 59(2):323–49, 1997.
